# Eating Habits and Lifestyle Factors Related to Childhood Obesity Among Children Aged 5-6 Years: Cluster Analysis of Panel Survey Data in Korea

**DOI:** 10.2196/51581

**Published:** 2024-04-05

**Authors:** Heemoon Lim, Hyejung Lee

**Affiliations:** 1 College of Nursing Yonsei University Seoul Republic of Korea; 2 Mo-Im Kim Nursing Research Institute College of Nursing Yonsei University Seoul Republic of Korea

**Keywords:** BMI, body mass index, childhood obesity, cluster analysis, healthy eating, healthy lifestyle, pediatric obesity, preschool child, prevention, unsupervised machine learning

## Abstract

**Background:**

Childhood obesity has emerged as a major health issue due to the rapid growth in the prevalence of obesity among young children worldwide. Establishing healthy eating habits and lifestyles in early childhood may help children gain appropriate weight and further improve their health outcomes later in life.

**Objective:**

This study aims to classify clusters of young children according to their eating habits and identify the features of each cluster as they relate to childhood obesity.

**Methods:**

A total of 1280 children were selected from the Panel Study on Korean Children. Data on their eating habits (eating speed, mealtime regularity, consistency of food amount, and balanced eating), sleep hours per day, outdoor activity hours per day, and BMI were obtained. We performed a cluster analysis on the children’s eating habits using *k*-means methods. We conducted ANOVA and chi-square analyses to identify differences in the children’s BMI, sleep hours, physical activity, and the characteristics of their parents and family by cluster.

**Results:**

At both ages (ages 5 and 6 years), we identified 4 clusters based on the children’s eating habits. Cluster 1 was characterized by a fast eating speed (fast eaters); cluster 2 by a slow eating speed (slow eaters); cluster 3 by irregular eating habits (poor eaters); and cluster 4 by a balanced diet, regular mealtimes, and consistent food amounts (healthy eaters). Slow eaters tended to have the lowest BMI (*P*<.001), and a low proportion had overweight and obesity at the age of 5 years (*P*=.03) and 1 year later (*P*=.005). There was a significant difference in sleep time (*P*=.01) and mother’s education level (*P*=.03) at the age of 5 years. Moreover, there was a significant difference in sleep time (*P*=.03) and the father’s education level (*P*=.02) at the age of 6 years.

**Conclusions:**

Efforts to establish healthy eating habits in early childhood may contribute to the prevention of obesity in children. Specifically, providing dietary guidance on a child’s eating speed can help prevent childhood obesity. This research suggests that lifestyle modification could be a viable target to decrease the risk of childhood obesity and promote the development of healthy children. Additionally, we propose that future studies examine long-term changes in obesity resulting from lifestyle modifications in children from families with low educational levels.

## Introduction

Childhood obesity has emerged as a major health issue due to the rapid growth in the prevalence of obesity among young children and the higher risk of developing cardiovascular and metabolic diseases in adulthood [1,2]. To address these health problems, childhood obesity has been studied for decades, and great efforts have been made to identify and characterize potential predictors of childhood obesity [3]. However, more studies are needed to understand the factors involved and their complex relationship with the development of childhood obesity [4].

Obesity can be caused by a combination of biological factors such as an individual’s genes, insulin resistance, disease, and metabolic processes, as well as socioeconomic factors such as the surrounding family and environment leading to obesity-related behaviors [5-7]. Although, fundamentally, excessive energy due to an imbalance between energy intake and consumed energy is known to cause fat formation and obesity, Davison and Birch [8] explain the various causes of childhood obesity as a micro- and macrosystem surrounding the child and provide evidence of the need for great efforts to change behavior to improve children’s health.

Eating habits affect dietary intake and obesity through various behaviors such as meal frequency, amount, speed, and snacking habits [9]. A prospective cohort study in which eating habits were measured repeatedly confirmed that there were individual differences in the development of food enjoyment and satiety responsiveness, which affect eating habits after the age of 4 years. These results suggest that eating habits are dynamic behaviors in the first years of life and may change beyond preschool age [10]. The GUSTO study measured the eating habits of children aged 5 and 6 years and found that obesity and overweight in children were related to rapid eating speed [11]. Therefore, understanding the early-life factors that influence these behaviors may help identify areas for intervention to curb the progression of being overweight or obese in children [12].

In terms of obesity prevention, the period of childhood before the age of 5 years is very important as an opportunity to establish new behaviors rather than change existing ones that have become entrenched in adulthood, which presents a difficult challenge [13]. Children’s eating habits begin with solid foods at the age of 3-6 months. From that time until the age of 5 years, preschool children learn autonomous eating habits from their parents and form eating habits based on their own preferences and previous experiences [14]. Additionally, preschool children aged 5 years or younger who are obese are more likely than children with a normal weight to become overweight during adolescence and are 5 times more likely to become obese as adults. Thus, prevention through healthy lifestyle habits early in childhood is important [15]. The importance of these early childhood lifestyle habits is highlighted by the World Health Organization’s guidelines for children’s health, which also discuss the importance of forming lifestyle habits in children before the age of 5 years [16].

Establishing a healthy lifestyle early in life is important to improve health outcomes later [17]. A recent literature review on childhood obesity revealed that, to prevent childhood obesity, changes need to be made in children’s overall lifestyle, including their daily living habits, rather than limiting management to food intake [18,19]. However, a number of studies generally recommend limiting the intake of high-calorie foods, sugary drinks, and fast foods and eating more fruit and vegetables to prevent childhood obesity [20-22]. As an eating practice guideline, dietary habits, such as eating breakfast, balanced eating, and eating slowly, are recommended during mealtimes, but studies on the relationship between these eating habits and early childhood obesity are limited [23,24]. Additionally, previous obesity research using machine learning explored the relationship between demographic factors, some behaviors, and childhood obesity but had limitations due to single cross-sectional methods and small sample sizes [25,26]. Therefore, this study is designed to identify characteristic patterns of preschool children’s eating habits using unsupervised machine learning techniques and to determine the impact of these eating habits on children’s BMI. Our results provide evidence that can guide healthy eating habits to prevent childhood obesity.

## Methods

### Study Design and Data

This study used data from the Panel Study on Korean Children (PSKC), which was designed to follow a sample of children from 2008 to 2027 to confirm the impact of families and communities on children’s growth and development. The PSKC is a nationally representative sample using stratified sampling that considers all the regions in South Korea. For this panel survey, parents with children born between April and July 2008 were recruited from 30 hospitals. In the first survey, a total of 2150 parents participated in face-to-face interviews and completed a self-administered questionnaire [27]. However, only 1280 children were included in this study because they had both the sixth and seventh surveys of the PSKC. For this study, the sixth data set (at the age of 5 years) and seventh data set (at the age of 6 years) were obtained for the data analysis after excluding missing and incomplete data (Multimedia Appendix 1). The data used for this study is considered a representative sample of national data in terms of the national demographics (male 649/1280, 50.7% and female 631/1280, 49.3%) and prevalence of childhood obesity (overweight 122/1280, 9.5% and obese 54/1280, 4.2%) [28].

### Measurements

#### Eating Habits

Eating habits were assessed based on four questions that mothers (or fathers) were asked to answer: “Is your child’s eating speed fast?” “Does your child have meals at regular times?” “Is the amount of food your child eats consistent?” and “Does your child eat all kinds of food?” Responses to each question were assessed using a 5-point Likert scale ranging from “not at all” to “agree very strongly.” A higher score indicated a greater tendency in the diet habit.

#### BMI

Obesity status, the primary outcome of this study, was defined according to BMI, which was calculated using the children’s weight and height [29]. The categories, such as normal, overweight, and obese, were defined based on the Korean child growth chart: children in the 85 to 95 percentile were categorized as overweight, and those over the 95 percentile were categorized as obese [30,31].

#### Physical Activity

For the children's activity levels, we used hours spent in outdoor activity as perceived by their mothers. They calculated the average number of hours their child spent daily on outdoor activities.

#### Sleep Duration

For the children’s sleep hours, we used the average amount of sleep time as perceived by their mothers. The child’s average sleep time at night was calculated as the difference between the mother’s reported bedtime and wake-up time.

#### Characteristics of Parents and Family

Parental age, education level, and employment status were obtained as parent characteristics; the number of family members and family income (Korean won per month) were obtained as family characteristics.

#### Ethical Considerations

This study was approved by the Hospital Ethics Committee, Seoul, South Korea (No. 4-2023-0418). The PSKC database was created with the voluntary consent of participants to investigate the growth and development of Korean children. If a participant decides to withdraw, they are excluded from the database. Digitally anonymized data sets were obtained after obtaining consent from PSKC in relation to the data. This study rigorously followed the guidelines recommended by the PSKC [32].

#### Statistical Analyses

All the continuous variables were tested for normality using the Shapiro-Wilk and Kolmogorov tests. The Shapiro-Wilk statistic was significant (*P*<.001), and the plots (regression of standardized residuals) showed no clear signs of violating the normality assumption [33].

**Figure 1:**
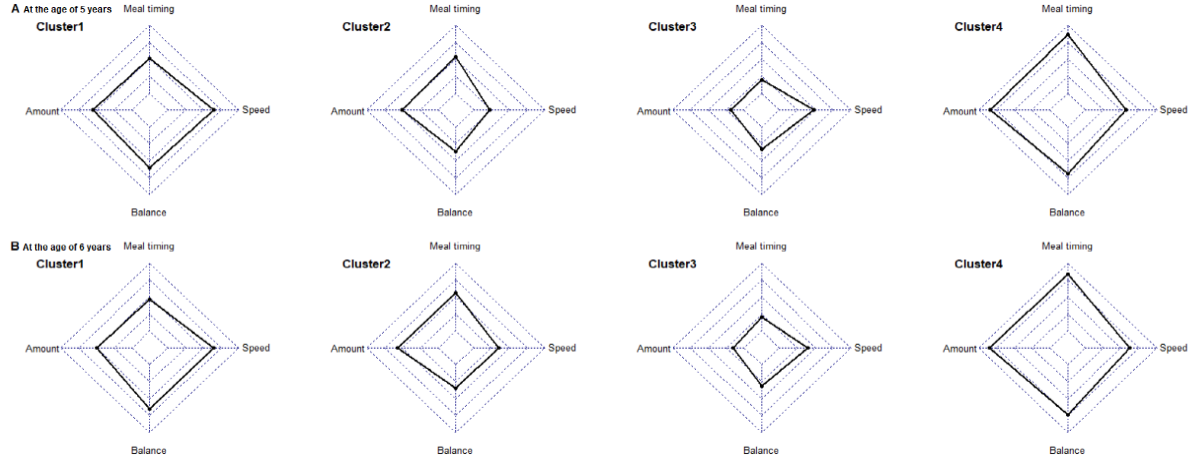
Characteristics of the clusters using a radar chart (A) at the age of 5 years and (B) at the age of 6 years. Individual eating patterns were clustered based on eating speed (speed), regularity of mealtime (meal timing), balanced eating (balanced), and consistency of meal amount (amount). The black line represents the average eating habits of that cluster.

Cluster analyses were performed in R (version 4.1.3; The R Project for Statistical Computing) using the packages “*tidyverse*,” “*cluster*,” “*factoextra*,” and “*NbClust*.” Clustering is an unsupervised machine learning technique to find natural groupings of participants based on a data set’s inherent structure. To identify the clusters, we used 4 eating habits (eating speed, mealtime regularity, food amount consistency, and balanced eating) as the input variables. For the ordinal variables measured on a 5-point Likert scale, we scaled by considering the means and SDs of the variables [26]. Principal component analysis was used to check the data distribution and independence of the 4 eating habits. Before the data set was considered significantly clusterable, the Hopkins statistic was applied iteratively using a threshold of 0.5, and the data set was confirmed to be above the threshold. The clustering analysis was performed by applying 2 hierarchical clustering methods (agglomeration and division) and Ward’s approach based on Euclidean distance and *k*-means [34]. We used the R *NbClust* package to explore the optimal number of clusters in our data set by varying all combinations of cluster number, distance measure, and clustering method and considered the Elbow method (*k*=4) and Scott index (*k*=4) for optimal cluster selection (Multimedia Appendix 2) [35]. Finally, the number of clusters (*k*=4) was selected by visually inspecting the data (clusters 1, 2, 3, and 4). After selecting the number of clusters, clusters were formed by repeating the *k*-means algorithm, which is most commonly used in unsupervised machine learning techniques, until the center value of the cluster did not change. Once the clusters were identified [35], a radar chart was created to explore the functionality of the final clusters (Figure 1).

A 1-way ANOVA with a Bonferroni posthoc comparison and a chi-square test were used to assess the group differences [36]. The power of this study was calculated using G*power (version 3.1) with a 95% degree of confidence, and the number of participants and study design were considered through a comparison of the differences between clusters. Statistical significance was defined as a 2-sided *P*<.05.

## Results

### Children’s Characteristics

Of the children analyzed, 50.7% (649/1280) were boys, and the mean birth weight was 3.26 (SD 0.41) kg. The average baseline BMI was 15.99 (1.60) kg/m^2^ at the age of 5 years and 16.20 (2.01) kg/m^2^ at the age of 6 years. At the age of 5 years, the children’s average time spent on outdoor activities was 1.14 (0.81) hours per day, and the average sleep duration per day was 9.87 (0.73) hours. The children’s average time spent on outdoor activities was 1.08 (0.71) hours per day, and the average sleep duration per day was 9.76 (0.68) hours at the age of 6 years.

A bachelor’s degree was the most prevalent education level for both mothers (485/1280, 37.89%) and fathers (552/1280, 43.13%). Most of the fathers (1226/1280, 96.09%) were employed, and the average family income was ₩4,275,100 (US $3186.64) per month (Multimedia Appendix 3).

### Cluster Developed by Eating Habits

The analysis identified 4 clusters, and their characteristics are similar on a radar chart (Figure 1). Cluster 1 (fast eaters) is characterized by a high eating speed and represents 512 children (aged 5 years) and 440 children (aged 6 years); cluster 2 (slow eaters) represents 293 five- and 415 six-year-old children with a slow eating speed. Cluster 3 (poor eaters) represents 283 children (aged 5 years) and 243 children (aged 6 years) with irregular mealtimes, inconsistent food amounts, and imbalanced eating habits, and cluster 4 (healthy eaters) represents 192 children (aged 5 years) and 182 children (aged 6 years) with regular mealtimes, consistent food amounts, and balanced eating (Table 1).

**Table 1:** Comparison of the children’s eating habits by cluster (N=1280).

Eating habit	At 5 years old, mean (SD)					At 6 years old, mean (SD)			
	Fast eater (n=512)	Slow eater (n=293)	Poor eater (n=283)	Healthy eater (n=192)	Fast eater (n=442)		Slow eater (n=415)	Poor eater (n=241)	Healthy eater (n=182)
Eating speed	3.24 (0.48)	1.94 (0.50)	2.72 (0.74)	3.01 (0.73)	3.47 (0.59)		2.48 (0.70)	2.60 (0.72)	3.40 (0.96)
Regularity of mealtime	3.91 (0.39)	3.97 (0.50)	2.98 (0.61)	4.98 (0.14)	3.95 (0.29)		4.19 (0.39)	3.25 (0.72)	4.92 (0.28)
Consistency of food amount	3.85 (0.38)	3.72 (0.50)	2.76 (0.53)	4.73 (0.45)	3.87 (0.36)		4.07 (0.32)	2.96 (0.60)	4.84 (0.37)
Balanced eating	3.65 (0.66)	2.76 (0.82)	2.65 (0.82)	4.00 (0.89)	4.03 (0.51)		2.79 (0.84)	2.69 (0.84)	4.38 (0.71)

### Cluster Changes From 5 to 6 Years of Age

Changes in clusters according to children’s eating habits are shown in Table 2. Of the total 1028 children, 553 (53.8%) remained in the same eating habits cluster as classified as being aged 5 years. A total of 44.7% (229/512) of children were in the fast eater group, 52.6% (154/293) in the slow eater group, 38.2% (108/283) in the poor eater group, and 32.3% (62/192) in the healthy eater group remained in the same cluster a year later.

**Table 2:** The cluster changes from 5 to 6 years of age. The bar graph represents the number of participants in the clusters at 5 years old who moved to another cluster at 6 years old.

Variable			Frequency, n/N (%)
**Fast eaters^a^**			
	Maintain	229/512 (44.7)	
	Slow eater	131/512 (25.6)	
	Poor eater	68/512 (13.3)	
	Healthy eater	84/512 (16.4)	
**Slow eaters^a^**			
	Maintain	154/293 (52.6)	
	Fast eater	66/293 (22.5)	
	Poor eater	52/293 (17.8)	
	Healthy eater	21/293 (7.2)	
**Poor eaters^a^**			
	Maintain	108/283 (38.2)	
	Fast eater	78/283 (27.6)	
	Slow eater	82/283 (29)	
	Healthy eater	15/283 (5.3)	
**Healthy eaters^a^**			
	Maintain	62/192 (32.3)	
	Fast eater	69/192 (35.9)	
	Slow eater	48/192 (25)	
	Poor eater	13/192 (6.8)	

^a^The cluster changes from 5 to 6 years of age in the relevant cluster.

### Characteristic Differences by Clusters at the Age of 5 Years

Among children’s characteristics, there was a significant difference in BMI at the age of 5 years between groups (*P*<.001). Fast eaters also had the highest BMI at the age of 5 years (mean 16.17 kg/m^2^); slow eaters had the lowest BMI (mean 15.59 kg/m^2^). The proportion of children with obesity differed significantly between groups (*P*=.03). A higher proportion of children with obesity, based on BMI at the age of 5 years, was reported among poor eaters (16/283, 5.7%) and fast eaters (28/512, 5.5%). There was a significant difference in sleep duration; fast eaters were associated with longer sleep duration (9.93 hours per day) than slow eaters (*P*=.005). Among parental and family characteristics, healthy eaters had a higher proportion of mothers with master’s degrees or higher, and poor eaters had a higher proportion of mothers who were high school graduates or lower (*P*=.03). There were no significant differences in the time children spent outdoors, family income, or parents’ employment status (Table 3).

**Table 3:** Differences in characteristics by cluster at the age of 5 years (N=1280).

Variable				Fast eater (n=512)		Slow eater (n=293)		Poor eater (n=283)	Healthy eater (n=192)		*P* value^a^			Posthoc test	
**Characteristics of child**															
	**Sex, n (%)**											.99			
		Male	261 (51)		146 (49.8)		145 (51.2)		97 (50.5)				N/A^b^		
		Female	251 (49)		147 (50.2)		138 (48.8)		95 (49.5)				N/A		
	Birth weight (kg), mean (SD)		3.28 (0.40)		3.22 (0.39)		3.26 (0.42)		3.28 (0.42)	.18			N/A		
	BMI at the age of 5 years (kg/m^2^), mean (SD)		16.17 (1.63)		15.59 (1.31)		16.03 (1.76)		16.06 (1.61)	<.001			a,c,d>b		
	Overweight, n (%)		53 (10.4)		21 (7.2)		25 (8.8)		23 (12)	.03			N/A		
	Obese, n (%)		28 (5.5)		4 (1.4)		16 (5.7)		6 (3.1)	N/A			N/A		
	Physical activity (hour/day), mean (SD)		1.16 (0.83)		1.17 (0.81)		1.06 (0.81)		1.13 (0.75)	.37			N/A		
	Sleep duration (hour/day), mean (SD)		9.93 (0.71)		9.79 (0.73)		9.79 (0.74)		9.93 (0.76)	.01			a>b		
**Characteristics of mother**															
	Age (years), mean (SD)		35.9 (3.46)		36.0 (3.43)		35.9 (3.74)		36.3 (3.42)	.77			N/A		
	**Education level, n (%)**											.03			N/A
		High school or less	152 (29.7)		78 (26.6)		94 (33.2)		50 (26)						
		College degree	132 (25.8)		96 (32.8)		78 (27.6)		50 (26)						
		Bachelor’s degree	210 (41)		99 (33.8)		100 (35.3)		76 (39.6)						
		Master’s degree or higher	18 (3.5)		20 (6.8)		11 (3.9)		16 (8.3)						
	**Employment status, n (%)**											.06			N/A
		Employed	206 (40.2)		124 (42.3)		111 (39.2)		97 (50.5)						
		Unemployed	306 (59.8)		169 (57.7)		172 (60.8)		95 (49.5)						
**Characteristics of father**															
	Age (years), mean (SD)		38.5 (3.97)		38.9 (3.70)		38.1 (4.0)		38.6 (3.79)	.10			N/A		
	**Education level, n (%)**											.07			N/A
		High school or less	146 (28.5)		74 (25.3)		83 (29.3)		44 (22.9)						
		College degree	100 (19.5)		62 (21.2)		65 (23)		28 (14.6)						
		Bachelor’s degree	213 (41.6)		136 (46.4)		105 (37.1)		98 (51)						
		Master’s degree or higher	53 (10.4)		21 (7.2)		30 (10.6)		22 (11.5)						
	**Employment status, n (%)**											.57			N/A
		Employed	489 (95.5)		284 (96.9)		268 (94.7)		185 (96.4)						
		Unemployed	23 (4.5)		9 (3.1)		15 (5.3)		7 (3.6)						
**Characteristics of family, mean (SD)**															
		Number of family members	4.29 (0.86)		4.27 (0.77)		4.20 (0.87)		4.25 (0.87)	.38			N/A		
		Income (₩10,000; US $7.45)	427.48 (227.85)		430.15 (173.44)		418.34 (211.31)		437.08 (175.79)	.79			N/A		

^a^*P* value was calculated from ANOVA and chi-square test.

^b^N/A: not applicable.

### Characteristic Differences by Clusters at the Age of 6 Years

Among children’s characteristics, there was a significant difference between groups in BMI at the age of 6 (*P*<.001). Fast eaters also had the highest BMI at the age of 6 years (mean 16.55 kg/m^2^); slow eaters had the lowest BMI (mean 15.85 kg/m^2^). The proportion of children with obesity differed significantly between groups (*P*=.01). A higher proportion of children with obesity, based on BMI at the age of 6 years, was reported among fast eaters (40/440, 9.1%) and healthy eaters (16/182, 8.8%). There was a significant difference in sleep duration; healthy eaters were associated with longer sleep duration (9.89 hours per day) than fast eaters (*P*=.03). Among parental and family characteristics, healthy eaters had a higher proportion of fathers with master’s degrees or higher, and poor eaters had a higher proportion of fathers who were high school graduates or lower (*P*=.03). There were no significant differences in the time children spent outdoors, family income, or parents’ employment status (Table 4).

**Table 4:** Differences in characteristics by cluster at the age of 6 years (N=1280).

Variable				Fast eater (n=440)		Slow eater (n=415)		Poor eater (n=243)		Healthy eater (n=182)		*P* value^a^		Posthoc test
**Characteristics of child**														
	**Sex, n (%)**											.24		
		Male	223 (50.7)		218 (52.5)		128 (52.7)		80 (44)				N/A^b^	
		Female	217 (49.3)		197 (47.5)		115 (47.3)		102 (56)				N/A	
	Birth weight (kg), mean (SD)		3.28 (0.39)		3.25 (0.40)		3.20 (0.41)		3.32 (0.45)		.02		d>c	
	BMI at the age of 6 years (kg/m^2^), mean (SD)		16.55 (2.11)		15.85 (1.74)		16.04 (2.01)		16.42 (2.17)		<.001		a>b,c; d>b	
	Overweight, n (%)		45 (10.2)		28 (6.7)		28 (11.5)		22 (12.1)		.005		N/A	
	Obese, n (%)		40 (9.1)		17 (4.1)		12 (4.9)		16 (8.8)		N/A		N/A	
	Physical activity (hour/day), mean (SD)		1.08 (0.69)		1.05 (0.69)		1.05 (0.74)		1.21 (0.75)		.07		N/A	
	Sleep duration (hour/day), mean (SD)		9.72 (0.67)		9.77 (0.69)		9.73 (0.69)		9.89 (0.68)		.03		d>a	
**Characteristics of mother**														
	Age (years), mean (SD)		35.8 (3.55)		36.0 (3.43)		35.9 (3.74)		36.3 (3.42)		.77		N/A	
	**Education level, n (%)**											.10		N/A
		High school or less	123 (28)		111 (26.7)		91 (37.4)		49 (26.9)					
		College degree	127 (28.9)		112 (27)		70 (28.8)		47 (25.7)					
		Bachelor’s degree	169 (38.4)		167 (40.2)		73 (30)		76 (41.8)					
		Master’s degree or higher	21 (4.8)		25 (6)		9 (3.7)		10 (5.5)					
	**Employment status, n (%)**											.21		N/A
		Employed	206 (40.2)		124 (42.3)		111 (39.2)		97 (50.5)					
		Unemployed	306 (59.8)		169 (57.7)		172 (60.8)		95 (49.5)					
**Characteristics of father**														
	Age (years), mean (SD)		38.5 (3.97)		38.9 (3.70)		38.1 (4.0)		38.6 (3.79)		.10		N/A	
	**Education level, n (%)**											.02		N/A
		High school or less	116 (26.4)		112 (27)		81 (33.3)		38 (20.9)					
		College degree	93 (21.1)		72 (17.3)		59 (24.3)		31 (17)					
		Bachelor’s degree	185 (42)		190 (45.8)		86 (35.4)		991(50)					
		Master’s degree or higher	46 (10.5)		41 (9.9)		17 (7)		22 (12.1)					
	**Employment status, n (%)**											.98		N/A
		Employed	489 (95.5)		284 (96.9)		268 (94.7)		185 (96.4)					
		Unemployed	23 (4.5)		9 (3.1)		15 (5.3)		7 (3.6)					
**Characteristics of family, mean (SD)**														
		Number of family members	4.26 (0.84)		4.25 (0.80)		4.28 (0.96)		4.30 (0.80)		.93		N/A	
		Income (₩10,000; US $7.45)	440.38 (229.69)		416.80 (165.07)		407.74 (239.91)		440.00 (169.47)		.08		N/A	

^a^*P* value was calculated from ANOVA and chi-square test.

^b^N/A: not applicable.

## Discussion

### Overview

Using a nationally representative sample, we identified 4 distinct clusters based on the eating habits of children aged 5 and 6 years. These children’s eating habits showed a pattern of 4 clusters a year later. However, in approximately half of the children, individual children changed their eating habit cluster after 1 year in this study, providing valuable insight into the timing of early obesity management [37]. Additionally, the higher proportion of children who had overweight and obesity and the higher BMI of children who ate quickly indicate that fast eating is associated with obesity. Children aged 5 years who are fast eaters need age-appropriate training to reduce their eating speed. Importantly, strategies to prevent progression from having overweight to having obesity in young children should be developed to improve children’s overall health status.

While a few studies have attempted to identify the relationship between eating habits and obesity in young children [4,23,38,39], none of them investigated eating habits concurrently, such as eating speed, balanced eating, mealtime regularity, and food amount consistency. In this study, we found that a large number of young children fell into the category of fast eaters and had a higher BMI. This relationship between fast eating habits and obesity can be explained by the mechanism that fast eating lowers satiety and consequently increases food intake by delaying the effects of brain signals and hormones [40]. The results of this study also suggest childhood obesity could be prevented by increasing eating time [39]. Regarding another eating habit related to childhood obesity, a recent systematic review identified mealtime as a mechanism that explains obesity by affecting changes in metabolic efficiency, hormones, and gut microbiota throughout the day [14]. Paoli et al [41] emphasized the importance of regular eating times for obesity prevention through the regularity of fasting periods between meals.

Regarding the relationship between sleep duration and childhood obesity, insufficient sleep could contribute to the development of obesity through appetite, diet, and daytime activity levels [42,43]. These studies did not support previous reports of late bedtimes and short sleep duration in children with obesity [44]. However, in this study, sleep duration per cluster was more than 9 hours, so all the children had enough sleep, which limits exploration of the relationship between sleep duration and obesity in this study. Additional research on sleep and childhood obesity is recommended.

Among the risk factors for childhood obesity, a more important explanatory factor is sedentary time, such as TV watching time, rather than outdoor activity [45]. Similarly, this study found no difference in outdoor activity levels between the groups classified based on eating habits. The relationship between childhood obesity and physical activity levels should be explored by considering various activities, such as sedentary time and indoor activities, in addition to outdoor activities.

Besides children’s sleep duration and activity levels, the parents’ education level differed among the clusters. The proportion of parents with a high school diploma or lower was higher in the group of poor eaters than in the other eating groups. These results are similar to those of previous studies showing that mothers of children with overweight or obesity had a lower education level [4]. This suggests that parents’ level of education is a factor related to the development of childhood obesity. However, the single influence of the mother or father by age, rather than both parents, is thought to account for differences in parental influence as the child ages and requires further research [46]. The prevalence of childhood obesity is known to be high in low-income households and communities with low socioeconomic status, and the relationship between childhood obesity and families’ economic status has been well documented in large samples in several countries [42,47,48]. However, family income in this study did not differ between the clusters, which suggests that primary caregiver education is the key factor in forming eating habits to consider for the prevention of obesity in children [43].

### Limitations

This study has several strengths and limitations that should be noted. Although the individual clusters had different BMI tendencies, the inclusion of only eating habit variables in the cluster analysis could limit our understanding of the development of childhood obesity since it omits interrelated variables such as physical activity and sleep. In addition, the results should be interpreted with caution because each eating habit was measured using a single item based on a Likert scale. However, we found evidence for the impact of dietary guidelines to prevent obesity in young children, including specific eating habits. Another limitation of this study was the reliance on parental reports. Nevertheless, the study is meaningful in that it used panel data that are representative longitudinal data of a country’s child population, and the derived results used machine learning techniques to solve complex phenomena targeting a relatively large sample of children.

### Conclusions

Our results show that eating habits, such as eating speed, regularity of mealtimes, meal amount consistency, and balanced eating habits, can be considered risk factors for developing childhood obesity. In addition, changed clusters by eating habits within 2 years in children highlights the need for early childhood obesity management. Besides eating habits, children’s sleep duration and maternal education levels differed significantly across the clusters. These findings suggest that a modification in lifestyle could be a good target to decrease the risk of childhood obesity and develop healthy children. In addition, we also propose that future studies examine long-term changes in obesity with lifestyle modification in children from families with low educational levels.

## Appendix

Multimedia Appendix 1 Flowchart of the process of selecting study participants. PSKC: Panel Study on Korean Children.

Multimedia Appendix 2 Evidence of clustering decision. (A) Result of the dendrogram at the age of 5 years; (B) Result of the dendrogram at the age of 6 years; (C) Results of choosing the number of clusters at the age of 5 years; (D) Results of selecting the number of clusters at the age of 6 years. (A) and (B) visually show the hierarchical method as one of the methods for selecting the number of clusters. (C) and (D) are the results of using the NbClust package of the R program and are the process for selecting the number of clusters through the D index and elbow method.

Multimedia Appendix 3 Characteristics of samples at the age of 5 and 6 years (N=1280).

## Abbreviations

PSKC: Panel Study on Korean Children

## Data Availability

The data sets generated and analyzed during this study are available from the corresponding author on reasonable request.
