# Supplementary figures and images for: Eating Habits and Lifestyle Factors Related to Childhood Obesity Among Children Aged 5-6 Years: Cluster Analysis of Panel Survey Data in Korea
Source: JMIR Public Health Surveill. 2024 Apr 5;10:e51581. doi: 10.2196/51581 (PMC11031700; doi:10.2196/51581)

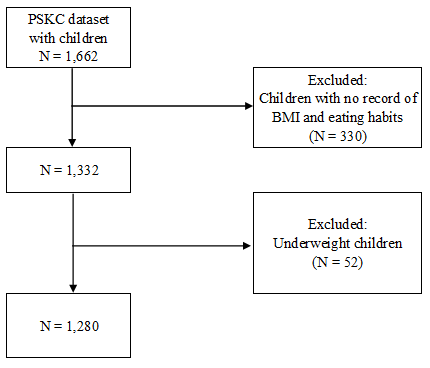

Supplement: Multimedia Appendix 1 [file publichealth_v10i1e51581_app1.png]

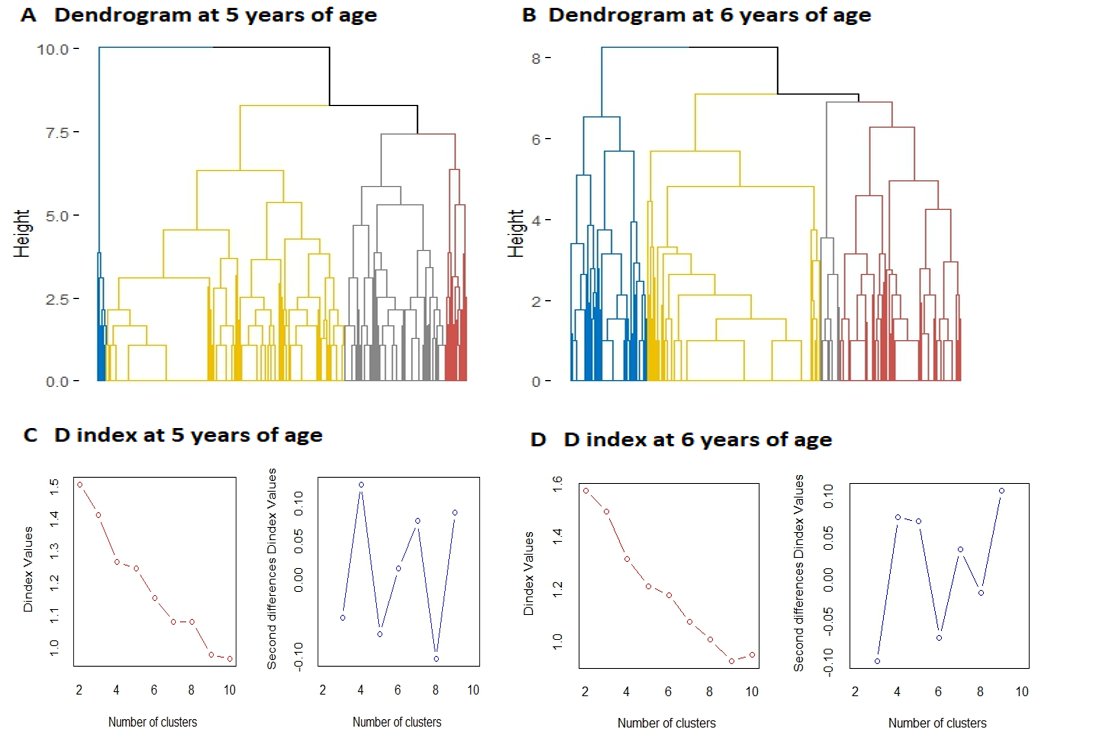

Supplement: Multimedia Appendix 2 [file publichealth_v10i1e51581_app2.png]
