# Supplementary material for: Eating Habits and Lifestyle Factors Related to Childhood Obesity Among Children Aged 5-6 Years: Cluster Analysis of Panel Survey Data in Korea
Source: JMIR Public Health Surveill. 2024 Apr 5;10:e51581. doi: 10.2196/51581 (PMC11031700; doi:10.2196/51581)
Supplement: Multimedia Appendix 3 [file publichealth_v10i1e51581_app3.docx]

**Table S1.** Characteristics of samples at the age of 5 and 6 years (N=1280).

| Characteristics | Mean | SD | Range |
| --- | --- | --- | --- |
| At five years old |  |  |  |
| Age (months) | 62.61 | 1.30 | 60–66 |
| Birth weight (kg) | 3.268 | 0.41 | 2–4.9 |
| Weight at 5 years old (kg) | 19.48 | 2.64 | 13.5–34.5 |
| Height at 5 years old (cm) | 110 | 4.31 | 97–128 |
| BMI at 5 years old (kg/m^2^) | 16.07 | 1.57 | 13.72–24.35 |
| Eating speed | 2.80 | 0.78 | 1–5 |
| Regularity of meal time | 3.88 | 0.75 | 1–5 |
| Consistency of food amount | 3.71 | 0.75 | 1–5 |
| Balanced eating | 3.28 | 0.94 | 1–5 |
| Physical activity (hours/day) | 1.14 | 0.81 | 0–5 |
| Sleep duration (hours/day) | 9.87 | 0.73 | 7–13 |
| Family income (10,000 won) | 428.3 | 207.42 | 0–3000 |
| At six years old |  |  |  |
| Age (months) | 75.10 | 1.39 | 72–79 |
| Weight at 6 years old (kg) | 22.41 | 3.62 | 15.4–38.8 |
| Height at 6 years old (cm) | 110 | 4.68 | 97–128 |
| BMI at 6 years old (kg/m^2^) | 16.27 | 2.01 | 12–30.85 |
| Eating speed | 2.98 | 0.85 | 1–5 |
| Regularity of meal time | 4.03 | 0.65 | 1–5 |
| Consistency of food amount | 3.90 | 0.68 | 1–5 |
| Balanced eating | 3.43 | 1 | 1–5 |
| Physical activity (hours/day) | 1.08 | 0.71 | 0–5 |
| Sleep duration (hours/day) | 9.76 | 0.68 | 7–13 |
| Family income (10,000 won) | 427.51 | 205.19 | 0–3000 |
